# Integration of poliovirus and enteropathogen sewage surveillance in Dhaka Bangladesh: a longitudinal surveillance study, June 2019–June 2020

**DOI:** 10.1016/j.lanmic.2025.101343

**Published:** 2026-05

**Authors:** Isobel M Blake, Md Ohedul Islam, Benjamin Fuller, Sarah Elwood, Suporn Pholwat, Jie Liu, Yoann Mira, A S G Faruque, Firdausi Qadri, Rashidul Haque, Mami Taniuchi

**Affiliations:** a MRC Centre for Global Infectious Disease Analysis, School of Public Health, Imperial College London, London, UK; b Department of Civil and Environmental Engineering, University of Virginia, Charlottesville, VA, USA; c Division of Infectious Diseases and International Health, University of Virginia, Charlottesville, VA, USA; d School of Public Health, Qingdao University, Qingdao, Shandong, China; e Novel-T, Geneva, Switzerland; f Nutrition Research Division, icddr,b, Dhaka, Bangladesh; g Infectious Diseases Division, icddr,b, Dhaka, Bangladesh; h Department of Biomedical Engineering, University of Virginia, Charlottesville, VA, USA

## Abstract

**Background:**

The Global Polio Eradication Initiative (GPEI) uses environmental surveillance to monitor circulation of poliovirus. After the certification of poliovirus eradication, environmental surveillance continues, generally through its integration into other infectious disease surveillance programmes. In this study, we evaluated the programmatic utility of TaqMan Array Cards (TACs) to detect poliovirus in sewage while simultaneously detecting enteric pathogens and markers of antimicrobial resistance (AMR).

**Methods:**

This longitudinal surveillance study was conducted across 12 sites in three wards (8, 9, and 10) in Dhaka, Bangladesh, from June, 2019, to June, 2020. Demographic data of children younger than 5 years, including vaccination history, sanitation, and household characteristics, were obtained from the Health and Demographic Surveillance System. Sewage samples were collected between June, 2019, and June, 2020, using a bag-mediated filtration system. We used TACs to detect poliovirus in sewage and simultaneously tested for other enteric pathogens and markers of AMR. Sites were selected after mapping of the informal sewage network and a demographic survey of the population. Samples were collected before and after a bivalent oral poliovirus vaccine (bOPV) campaign. We used a water-quality probe to assess physicochemical properties of the sewage. A multivariable mixed-effects gamma hurdle regression model was used to examine the association of enterovirus detection and concentration with site properties.

**Findings:**

The Health and Demographic Surveillance System recorded 19 236 children younger than 5 years across 12 sites in the three wards in Dhaka. The bOPV campaign reached 17 282 children. 372 sewage samples were collected over 379 days. The highest concentration of Sabin 1 and Sabin 3 polioviruses was detected 2 weeks after the bOPV campaign (mean viral copies per litre of sewage were 0·83 [SD 2·13] for Sabin 1 and 0·84 [1·96] for Sabin 3 *vs* baseline values of 0·05 [0·21] for Sabin 1 and 0·11 [0·50] for Sabin 3; p=0·004 for Sabin 1 and p=0·005 for Sabin 3). Detection of enteroviruses was more likely with increasing levels of total dissolved solids (adjusted odds ratio per absolute increase of 100 mg/L 1·39 [95% CI 1·17–1·61]). The median environmental surveillance viral load of rotavirus was 0·567 (IQR 0·202–0·839), and rotavirus had the strongest correlation with respective concurrent clinical case incidence (r=0·828; p=0·0017). 31 AMR genes of clinical significance were detected.

**Interpretation:**

When the GPEI dissolves, poliovirus surveillance would need to be integrated into other surveillance programmes. TACs could be used to screen suitable pathogens for integrated sewage surveillance alongside poliovirus. Further validation is required across different geographies and poliovirus prevalence, and data interpretation requires an understanding of site sensitivity.

**Funding:**

Gates Foundation.

## Introduction

Environmental surveillance for poliovirus is an important complementary surveillance system used by the Global Polio Eradication Initiative (GPEI). The gold standard surveillance approach involves reporting cases of acute flaccid paralysis (one to three per 100 000 children younger than 15 years) and testing stool samples from these cases for poliovirus.^1^ However, most cases of poliovirus infections are asymptomatic,^2^ and environmental surveillance provides an approach to capture an early warning of transmission and silent circulation.^3^ Both wild and oral poliovirus vaccine (OPV)-related polioviruses are shed in the stool for 4–8 weeks,^4^ and poliovirus does not persist in the environment,^5^^,^^6^ meaning the virus detection in sewage is indicative of recent transmission events. By the end of 2023, systematic testing of sewage was established in 86 countries worldwide.^7^ Outbreaks of wild poliovirus and vaccine-derived poliovirus (VDPV) have been identified through environmental surveillance,^3^ and this surveillance method is crucial to the GPEI during the polio endgame.^8^

**Research in context**

**Evidence before this study**

Environmental surveillance, particularly wastewater and sewage surveillance, is used as a supplementary surveillance method by the Global Polio Eradication Initiative (GPEI) and was rapidly expanded across multiple countries during the COVID-19 pandemic to monitor SARS-CoV-2. Following polio eradication, environmental surveillance for poliovirus will need to be integrated with surveillance for other infectious diseases to ensure cost-effectiveness, and optimal approaches for such integration need to be identified. We searched PubMed from database inception to Dec 15, 2024, without language restrictions, using the combined search terms “poliovirus”, “environmental surveillance”, “enteric pathogens”, and “antimicrobial resistance”. The search yielded no results. Although we did not find any current literature regarding the integration of multipathogen surveillance into an existing poliovirus network, several studies addressed the detection of pathogens of interest in wastewater surveillance. A previous study reported the use of environmental surveillance in the detection of enteric pathogens with outbreak potential. Furthermore, previous studies showed that antimicrobial resistance (AMR) genes can be detected in wastewater, but the clinical impact of detecting AMR genes in sewage remains unclear. The vast majority of previous environmental surveillance studies has been done in high-income countries with established formal sewage networks, with the exception of the polio environmental surveillance use case.

**Added value of this study**

Sewage surveillance for poliovirus within an integrated framework to test for other enteric pathogens and markers of AMR has not previously been described in high-income settings with established sewage networks, much less in low-resource settings with an informal converging sewage network. In this study, we showed the feasibility of using TaqMan Array Card (TAC) technology for integrated sewage surveillance in Dhaka, Bangladesh. By incorporating 15 traditional poliovirus targets with 51 additional targets for enteric pathogens and AMR genes, a more comprehensive picture of pathogen circulation and resistance patterns can be obtained. The inclusion of a broad array of pathogens and AMR genes supports the goal of sustaining poliovirus surveillance while expanding the use of sewage surveillance to inform a wider range of public health threats. Vaccine poliovirus was detected in high concentration following a vaccination campaign, showing its potential as a platform for integrated surveillance. Enteric pathogen detection was compared with concurrent clinical surveillance.

**Implications of all the available evidence**

For poliovirus surveillance to be sustainable in the post-eradication era, it should be integrated with surveillance for other pathogens. Our findings show that the integration of wastewater surveillance for multiple pathogens into systematic environmental surveillance is feasible. Concurrent clinical data are crucial to validate and appraise the public health implications of wastewater surveillance data. Further validation in different populations is urgently needed, especially to assess TAC sensitivity in populations with low poliovirus prevalence, given the GPEI's goal to certify polio eradication by 2027.

The GPEI will dissolve after the certification of eradication of type 1 wild poliovirus and elimination of VDPV. However, surveillance for poliovirus will still be required given the continued risks of poliovirus circulation arising from either undetected vaccine-derived circulation, exposure from chronic VDPV excretion from immunodeficient individuals, illicit use of OPV, or viral escape from laboratories (containment failure).^3^ Evidence of some of these risks has occurred after the global withdrawal of type 2 polioviruses from OPV.^9^ When the GPEI dissolves, funds for surveillance activities will be greatly reduced. The GPEI Post Certification Strategy plans for environmental surveillance to be a fundamental core surveillance approach in this future era. Integration of environmental surveillance into other sewage surveillance activities, such as monitoring for other pathogens or antibiotic resistance, will allow environmental surveillance for poliovirus to be more cost-effective, enabling continued surveillance for poliovirus across larger populations;^3^ however, integrated multipathogen environmental surveillance, including that for poliovirus, has not been established to date.^7^

TaqMan Array Cards (TACs) allow for simultaneous detection of multiple pathogens from a single environmental surveillance or clinical sample. This technology has been used in multiple international epidemiological studies in low-income and lower-middle-income countries to identify the cause of diarrhoea in children and evaluate the interference of enteropathogens with the effectiveness of OPV and rotavirus vaccines.^10^, ^11^, ^12^ The utility of TAC has been shown as a high-throughput tool for detecting multiple pathogens in environmental samples such as wastewater from high-income settings.^13^

Effective laboratory algorithms to simultaneously test for multiple targets in sewage alongside poliovirus have not yet been developed but are essential for successful integrated sewage surveillance. In this study, we evaluated the programmatic utility of TACs to detect poliovirus in sewage while simultaneously detecting other enteric pathogens and markers of antimicrobial resistance (AMR). We assessed the use of TACs to detect Sabin 1 and Sabin 3 polioviruses (OPV strains) after a mass vaccination campaign with bivalent OPV (bOPV) in Dhaka, Bangladesh.

## Methods

This longitudinal surveillance study was conducted in Dhaka, Bangladesh (a study area of 7·8 km^2^), from June, 2019, to June, 2020. Detailed demographic and sewage network data were collected within 7 months before commencing the longitudinal study to inform the choice of sampling sites.

### Demographic and sewage network data collection

Detailed demographic information from children younger than 5 years was collected from the Health and Demographic Surveillance System,^14^ initiated at the start of the study period. Recorded data included age, number of OPV and inactivated poliovirus vaccine doses received, household size and location, antibiotic use, sanitation level, and socioeconomic status of each individual child.

The study team mapped the location and direction of sewage flow by walking along every sewage channel, tracing the informal and formal sewage lines onto a physical map. These sewage lines were subsequently digitised using QGIS to create shapefiles.

### Site selection

Watershed areas of candidate sites were estimated using the mapped sewage network data and digital elevation models at 2-m resolution. Candidate environmental surveillance sites were evaluated through summing the child population residing within candidate site watersheds. Population estimates from WorldPop were used to estimate the population size residing within watersheds outside the study area, assuming that the children younger than 5 years account for 9% of the total population, based on the proportion observed in the Health and Demographic Surveillance System in the study area. The final sampling locations were based on the catchment population and accessibility to the site. Four sites were selected in each of the wards 8, 9, and 10 in Dhaka, equating to 12 sites in total.

The study protocol was approved by the research review committee and ethical review committee of the International Centre for Diarrhoeal Disease Research, Bangladesh (icddr,b), and the institutional review board of the University of Virginia. Written informed consent was obtained from the parents or legal guardians before vaccinating each child under 5 years of age in the study area. This study is registered with ClinicalTrials.gov (NCT03818477).

### Sample collection and processing

Environmental surveillance samples were collected between June 9, 2019, and June 22, 2020. Samples were collected at weekly intervals during the month before the bOPV campaign, every 3 days during the month after the campaign, weekly for the subsequent 2 months, and monthly for the remainder of the study (except for April, 2020, owing to COVID-19-related national lockdown; appendix p 4). 6 L of sewage samples were collected between 0700 h and 0930 h using the bag-mediated filtration system^15^ and filtered through ViroCap filters at the field office. Subsequently, the ViroCap filter housing was placed on ice packs to maintain cold chain during transport to the icddr,b laboratory for pathogen elution, concentration, and nucleic acid extraction. Details of the field and laboratory procedures are provided in the appendix (p 3). The total nucleic acid was stored at –80°C until further testing.

At the time of sampling, physicochemical properties of the sewage samples were assessed using Aquaprobe AP-2000 devices (Aquaread, Broadstairs, UK), including temperature, barometric pressure, pH, oxidation–reduction potential, dissolved oxygen, total dissolved solids (TDSs), salinity, and altitude, according to a protocol developed previously.^16^ Duplicate readings were taken at each time of sampling, and the mean of the two readings was noted for each measurement.

### Environmental surveillance using TACs

The customised TACs for environmental surveillance included 66 targets in total, of which 15 were enteroviruses, including poliovirus; 36, AMR genes; 13, non-enterovirus enteric pathogens, and two, extrinsic controls, MS2 and PhHV (appendix pp 5–7). The pan-enterovirus assay captured all enteroviruses, including poliovirus.^17^ For some pathogens, multiple targets were used. The assay for *Vibrio cholerae* captured all serogroups. All detections with a cycle threshold greater than or equal to 35 were considered negative, with the exception of poliovirus targets where the cycle threshold was 36 for Sabin 1 and 37 for Sabin 3.^18^ Valid results required positive extrinsic controls for negative samples, absence of contamination in extraction blanks, and PCR-run analysis results to be free of quality-control flags. The pathogen and AMR gene targets tested using TACs in this study are listed in the appendix (pp 5–7). We estimated the pathogen load per litre of the filtered environmental sample. Because each grab of sewage yielded a different volume of filtered sample using the bag-mediated filtration system, we calculated the number of copies per litre of filtered sewage. We present pathogen load estimates using the following scale: log_10_ ([total sample pathogen load/volume of filtered sewage sample] + 1), where + 1 is added to keep the test negatives at 0 on the log scale.

### Vaccination campaign with bOPV

Children younger than 5 years were vaccinated with bOPV during June 29–July 1, 2019, in ward 8; July 2–4, 2019, in ward 9; and July 6–8, 2019, in ward 10. Vaccines were administered at multiple vaccine centres, which were strategically placed throughout the wards such that the majority of families with children could access a vaccine centre within 400 m from their homes. Mobile units for vaccine delivery were used to deliver vaccines to children who lived further than 400 m from a vaccine centre. Vaccination coverage was assessed by calculating the percentage of children vaccinated with respect to the total population younger than 5 years in the study area.

### Hospital-based diarrhoea surveillance data

We used clinical surveillance data obtained from icddr,b. In short, more than 150 000 patients with diarrhoea visit icddr,b per year, and stool samples from 2% of all patients with diarrhoea seeking hospital care are systematically tested for rotavirus and enterotoxigenic *Escherichia coli* using PCR and for *V cholerae*, *Shigella* spp, and *Salmonella* spp using culture after receiving consent from patients or parents or legal guardians of children.^19^ Notably, during 2020, the number of patients having diarrhoea who visited the hospital during the COVID-19 lockdown reduced, meaning the 2% surveillance data potentially underassessed clinical burden during this period.

### Statistical analysis

One-way ANOVA was used to assess whether variation in physicochemical sewage measurements was greater between sites than within sites. The Wilcoxon rank-summed test was used to identify whether the mean of physicochemical measurements was greater in some locations than others.

Markers of site sensitivity were evaluated by fitting a mixed-effects gamma hurdle regression model to the number of viral copies of enterovirus per litre of filtered sewage. A site-level random effect was allowed in both the hurdle model and the conditional gamma model. A full set of covariates, comprising physicochemical properties of the sewage sample and site demographics (eg, estimated site catchment area, population size, vaccination history, and level of sanitation), is given in the appendix (p 8). We also allowed a categorical variable indicating whether the sample was collected within 3 weeks of the bOPV campaign. Univariable analyses were performed within the hurdle or gamma model separately. The Akaike information criterion (AIC) of the full multivariable model (ie, the model containing all covariates in the hurdle and conditional gamma models) was evaluated. To identify the most simple yet best fitting model, backwards multivariable model selection was performed, removing variables in a stepwise manner and retaining those that minimised the AIC. Models were built using the glmmTMB package (version 1.1.13) in the R programming language.^20^

We compared monthly clinical diarrhoeal incidence of rotavirus, enterotoxigenic *E coli*, *V cholerae*, and *Shigella* spp among individuals seeking care at icddr,b and residing in Dhaka with the corresponding mean quantitative detection of these pathogens in sewage during the study period. The linear relationship between pathogen load in sewage and clinical case count was assessed by calculating Pearson correlation coefficients. Measuring the correlation over 11 monthly observations provided 89% power to detect a strong correlation (*r*>0·8) but only 24–53% power to detect a moderate correlation (*r*=0·4–0·6), assuming an α significance level of 0·05. Enteric pathogen and AMR analyses were performed using R (version 4.4.1).

### Role of the funding source

The funders of the study had no role in study design, data collection, data analysis, data interpretation, or writing of the report.

## Results

**Figure 1:**
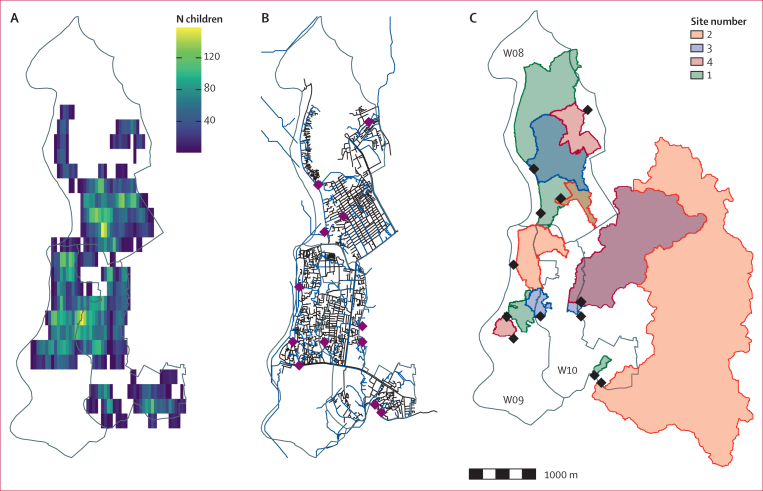
Geospatial demographic and sewage characteristics of wards 8, 9, and 10 in Dhaka, Bangladesh (A) Population density of children younger than 5 years (white areas indicate no children living in that area). (B) Sewage channels (black) and estimated drainage lines (blue); the selected sampling sites are shown as purple diamonds. (C) Estimated watershed areas of each site (note that some overlap and so the colours of the catchments reflect the overlap [eg, sites 3 and 4 in W10 and sites 1 and 2 in W08]). Grey lines indicate ward boundaries, and sampling sites are shown as black diamonds.

The Health and Demographic Surveillance System recorded 19 236 children younger than 5 years residing in wards 8, 9, and 10 among 8739 households. The average child population density across the area was 2466 children per km^2^, although the distribution of children was heterogeneous across the study area (figure 1A). 1831 sewage channels were mapped (figure 1B). The estimated area of each watershed site ranged between 0·04 km^2^ and 7 km^2^ (figure 1C), and the estimated catchment populations of the 12 sites ranged between 195 and 58 208 children (table 1), with three sites in ward 10 possibly receiving sewage from children outside the study area. The number of children with recorded OPV vaccination histories was 7747 (40·3%) of 19 236, and the proportion of children within each catchment reporting having received three OPV doses before the study varied between 0·68 and 0·91 (table 1).

**Table 1:** Environmental site characteristics in wards 8, 9, and 10 of Dhaka, Bangladesh

	Estimated watershed area (km^2^)	Estimated catchment population younger than 5 years residing in the study area (n)	Proportion of children in each catchment with reported vaccination history	Proportion of children in each catchment with vaccination history reporting three OPV doses	Proportion of children in each catchment with a sanitary toilet (without flush)	Proportion of children in each catchment with a sanitary toilet (with flush)
Ward 8, site 1	2·12	4461	0·43	0·76	0·86	0·10
Ward 8, site 2	0·14	632	0·41	0·76	0·86	0·12
Ward 8, site 3	0·71	1413	0·45	0·76	0·90	0·06
Ward 8, site 4	0·31	307	0·55	0·68	0·98	0·01
Ward 9, site 1	0·14	990	0·43	0·74	0·96	0·04
Ward 9, site 2	0·51	2142	0·37	0·80	0·95	0·02
Ward 9, site 3	0·12	1175	0·29	0·80	0·98	0·02
Ward 9, site 4	0·08	482	0·42	0·82	0·98	0·02
Ward 10, site 1	0·04	195	0·31	0·84	0·99	0·00
Ward 10, site 2	7·01	58 208	0·42	0·91	0·52	0·48
Ward 10, site 3	2·13	18 396	0·49	0·82	0·81	0·03
Ward 10, site 4	2·09	18 212	0·51	0·81	0·78	0·02

Vaccination coverage and sanitation variables were collected through the Health and Demographic Surveillance System. OPV=oral poliovirus vaccine.

**Figure 2:**
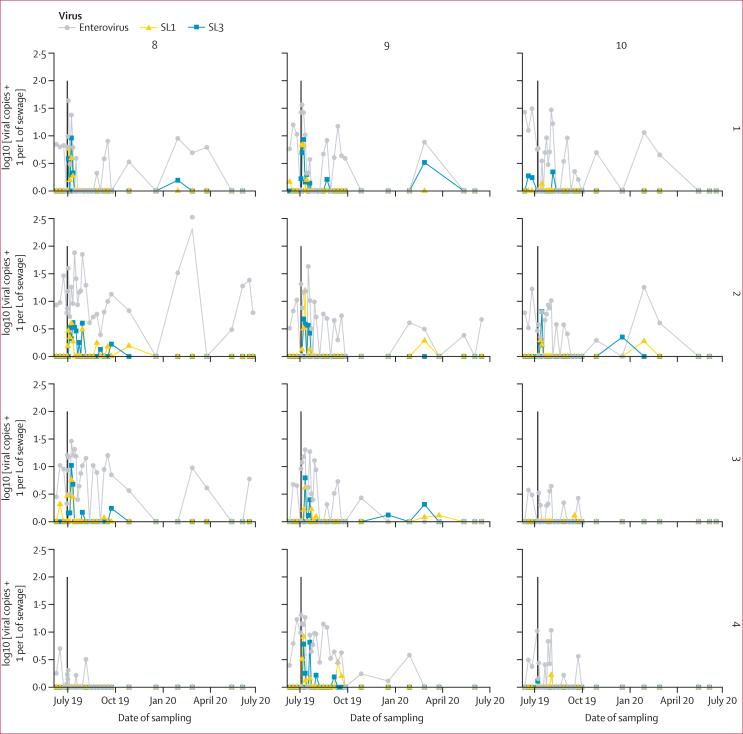
Viral load of Sabin 1 (SL1) and Sabin 3 (SL3) polioviruses detected in sewage samples collected from wards 8, 9, and 10 in Dhaka, Bangladesh, from four sites within each ward The timing of the mass immunisation campaign with bivalent oral poliovirus vaccine (containing Sabin 1 and Sabin 3 viruses) is shown by the black line. Viral load of enterovirus is given in grey (this includes polioviruses and non-polioviruses).

The bOPV vaccination campaign reached 17 282 children younger than 5 years in the study area (89·8%). In total, 372 sewage samples were collected across the study period over 379 days. The number of samples collected from each site per month is given in the appendix (p 4). Sabin vaccine virus was detected in 11 (92%) of 12 sites during the study period. The highest concentration of Sabin 1 and Sabin 3 was detected 2 weeks after the bOPV campaign (figure 2; mean viral copy number per litre of sewage was 0·83 [SD 2·13] for Sabin 1 and 0·84 [1·96] for Sabin 3 *vs* baseline mean values of 0·05 [0·21] for Sabin 1 and 0·11 [0·50] for Sabin 3; p=0·004 for Sabin 1 and p=0·005 for Sabin 3; Wilcoxon rank-summed test); however, variation in the detection of poliovirus across the sites was evident. The concentration of enteroviruses was higher than that of Sabin virus across the study period (mean viral copy number was 0·48 [0·50]) and fluctuated by month, with wide variation across the sites (figure 2). No wild poliovirus, VDPV, or any type 2 polioviruses were detected during the study.

Physicochemical measurements of the sewage varied both across sites and over time (appendix p 9). Higher variation was observed between sites than within sites for all measured variables (p<0·001), except for sewage temperature (p=0·68) and resistivity (p=0·18), which showed temporal variation across sites. The sewage temperature was seasonal, reaching a low of 22·2°C in January, 2020, and 31·2°C in August, 2019 (appendix p 9). TDS in sites from ward 9 was, on an average, higher than that recorded in the other two wards (p<0·001), whereas pH was notably lower in site 4 of ward 8 than that in the other sites (p<0·001).

Considering the variations observed in Sabin vaccine poliovirus detection across sites, we investigated covariates of site sensitivity. As the detection of the vaccine virus was rare across the complete time series, we assessed covariates associated with enterovirus detection, as the enterovirus detection rate is used by the GPEI to measure site performance. Univariable results of covariates associated with enterovirus detection in sewage are given in the appendix (p 8). In summary, demographic and vaccination variations by site (table 1) were not significantly associated with enterovirus detection, whereas some physicochemical site properties were significantly associated with enterovirus detection. Based on the final mixed-effects multivariable model (AIC=1680·7, ΔAIC=8 from the full model), enteroviruses were more likely to be detected from environmental surveillance samples with increasing levels of TDS (table 2), such that an increase of 100 mg/L of TDS (an indirect measure of sewage concentration) was associated with the odds of detecting enteroviruses being 1·39 times higher than baseline (95% CI 1·17–1·61), adjusting for sewage temperature and the additional Sabin viruses excreted within the 3 weeks after the bOPV campaign. Given the sample was positive, the viral load was positively associated with the oxidation–reduction potential of the sample (ie, with increasing aerobic conditions) and negatively associated with sewage temperature, adjusting for the additional Sabin viruses excreted within the 3 weeks after the bOPV campaign. There was no evidence for outliers or overdispersion, and model residuals were uniformly distributed (appendix p 10).

**Table 2:** Multivariable mixed-effects gamma hurdle regression model of enterovirus detection in environmental surveillance sewage samples collected from Dhaka, Bangladesh

	Odds ratio (95% CI) associated with a negative sample	Rate ratio (95% CI) of viral load, conditional on the sample being positive
Total dissolved solids, per absolute increase of 100 mg/L	0·72 (0·62–0·85)	··
Oxidation–reduction potential, per absolute increase of 100 mV	··	1·68 (1·27–2·24)
Sewage temperature, per degree increase	0·79 (0·68–0·92)	0·90 (0·83–0·98)
First 21 days after the immunisation campaign with the bivalent oral poliovirus vaccine *vs* the rest of the study period	0·52 (0·30–0·92)	1·48 (1·12–1·94)
Variance of the site-level random effect	0·85	0·44
σ^2^ (gamma distribution)	··	0·68

**Figure 3:**
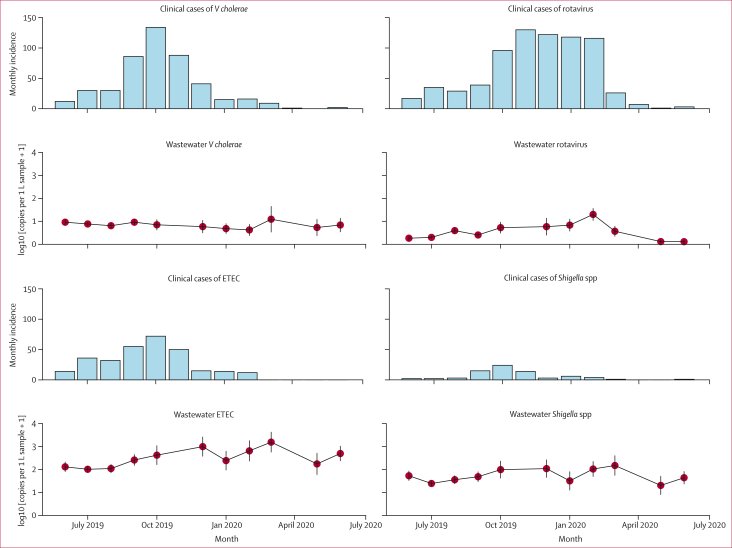
Monthly mean concentration of enteric pathogens from sewage samples collected in wards 8, 9, and 10 in Dhaka, Bangladesh Error bars denote 95% CI values around the mean, alongside respective clinical data from the International Centre for Diarrhoeal Disease Research, Bangladesh, from June, 2019, to July, 2020. ETEC=enterotoxigenic *Escherichia coli*.

The median pathogen load of pan-*V cholerae* detected in sewage was 0·889 copies per 1 L of sewage (IQR 0·633–1·19), that of rotavirus was 0·567 copies per 1 L of sewage (0·202–0·839), and that of *Shigella* spp was 1·791 copies per 1 L of sewage (1·24–2·131; figure 3). Enterotoxigenic *E coli* was detected at considerably higher levels than other pathogens throughout the study period, with a median pathogen load of 2·415 copies per 1 L of sewage (IQR 1·913–2·789). Only monthly mean rotavirus concentration in sewage was statistically correlated with monthly clinical incidence (r=0·828; p=0·0017; appendix p 8). The mean rotavirus load in sewage was the highest from November to February (winter), at 0·967 copies per 1 L of sewage (SD 0·291), compared with that from March to October, at 0·386 copies per 1 L of sewage (0·267), which corresponded to higher mean monthly clinical cases during the winter versus summer months (118·7 [25·1] *vs* 31·6[27·4]; figure 3). Additional enteric pathogens were detected during the study period, but no concurrent clinical data were available to assess a correlation. Information regarding the detection of additional pathogens without corresponding clinical data during the study period that were reliably detected (>2 sequential detections) can be found in the appendix (pp 10–11).

Multiple clinically important AMR genes that confer resistance to β-lactams, quinolones, macrolides, and polymyxins were detected in sewage. Of the 372 samples collected, *bla*_OXA48_ was detected in 349 (93·8%) of samples, *bla*_VIM_ in 258 (69·4%), *bla*_NDM_ in 304 (81·7%), and *bla*_KPC_ in 360 (96·8%), with median concentrations of 1·52 copies per 1 L of sewage (IQR 0·99–1·9) for *bla*_OXA48_, 0·42 copies per 1 L of sewage (0–0·94) for *bla*_VIM_, 1·24 copies per 1 L of sewage (0·51–1·74) for *bla*_NDM_, and 1·25 copies per 1 L of sewage (0·71–1·83) for *bla*_KPC_. *qepA* and *qnrA* were clinically relevant AMR genes conferring resistance against quinolones; *qepA* was detected in 258 (69·4%) of 372 samples and *qnrA* in 311 (83·6%) of samples, with median concentrations of 0·41 copies per 1 L of sewage (IQR 0–0·86) for *qepA* and 0·54 copies per 1 L of sewage (0·24–0·84) for *qnrA*. *ermB*, *mcr-1*, and *mphA* were AMR genes conferring resistance against macrolides and polymyxins. *ermB* was detected in 94·1% of samples, *mcr-1* in 88·4%, and *mphA* in 100%, with median concentrations of 2·57 copies per 1 L of sewage (IQR 2·1–2·83) for *ermB*, 0·65 copies per 1 L of sewage (0·34–0·99) for *mcr-1*, and 3·88 copies per 1 L of sewage (3·53–4·10) for *mphA*. Additional information regarding the full list of AMR gene targets is provided in the appendix (pp 5–7).

## Discussion

Eradication of wild-type poliovirus is in its final stages, with endemic transmission remaining in two countries: Pakistan and Afghanistan. Implementation of direct detection methods for environmental surveillance would allow for timely detection of ongoing circulation of poliovirus.^7^ Furthermore, following eradication, direct detection is essential given the risk of exposure from silent circulation, immunodeficient individuals chronically excreting VDPV, and risk from laboratory escape.^21^ Given the strategy of the GPEI to certify wild poliovirus eradication in 2028,^22^ efficient systems to conduct surveillance in the post-eradication era must be designed now in advance of this date. Sewage surveillance is an efficient non-invasive method to anonymously sample from large populations in urban areas, and its sustainability for poliovirus surveillance will be increased if it can be integrated into surveillance for other pathogens.^23^ We showed that TACs can successfully detect Sabin poliovirus following a mass bOPV campaign with high coverage, alongside detecting other enteric pathogens and markers of AMR, and might therefore provide a platform to screen a broad range of pathogens suitable for deciding what panel of pathogens would be suitable for integrated sewage surveillance (following further validation against current methods from the Global Polio Laboratory Network).

We also showed variation in the detection of poliovirus following the bOPV campaign across environmental surveillance sites. Three of the environmental surveillance site catchments in ward 10 largely fell outside the study area where the bOPV campaign was not implemented, resulting in minimal detection of Sabin virus at these sites. Sensitivity of environmental surveillance to detect poliovirus is often measured by quantifying the sensitivity of general enterovirus detection, given the higher prevalence of enterovirus than that of poliovirus. We found that enterovirus detection varied across sites and that TDS measured in the sewage at the time of collection was associated with the ability to detect enterovirus. This finding complements the finding of a previous study in Nigeria that reported an association between TDS and enterovirus detection.^16^ High TDS likely reflects the concentration of faecal contamination in the sewage and could provide a more accurate measure of the population represented by the sewage sample at a given time than our static estimates of the site catchment. This finding provides further evidence that monitoring the physicochemical properties of sewage can benefit the GPEI to help with identifying sensitive environmental surveillance sites. Site catchment area, population size, vaccination history, and sanitation level did not sufficiently explain the variation across sites. The need for a site-level random effect in the model indicates unexplained variation across sites, and further research is needed to fully understand environmental surveillance site-level variation.

We also found temporal variation in the pathogen load of several enteric pathogens across the 1-year study period and observed a significant correlation between the seasonal trend of rotavirus obtained from diarrhoea-related hospitalisations in Dhaka (in agreement with sewage surveillance for rotavirus in other populations).^24^^,^^25^ Specifically, monthly mean rotavirus concentrations in sewage were strongly correlated with monthly diarrhoea-related hospitalisations in Dhaka, aligning with findings from sewage surveillance in other populations. Rotavirus loads in sewage were the highest during the winter months (November–February) and substantially lower during the summer months (March–October). This pattern mirrors seasonal trends in clinical cases.

Although enterotoxigenic *E coli* was detected at considerably higher concentrations than any other pathogen in sewage throughout the study period, it was not among the leading causes of hospitalisations. In contrast, rotavirus and *V cholerae* had the highest number of clinical cases, despite showing lower median sewage concentrations. *Shigella* spp, another major pathogen within Dhaka, had a comparatively high sewage signal, although the clinical case burden was not as substantial.

The observed discordance between clinical and environmental surveillance systems can be attributed to several factors. First, pathogens exhibit variable shedding rates and durations, influencing their detectability in sewage. Second, the severity of illness varies by pathogen, weakening the correspondence between sewage observations and clinical reports. Third, pathogen-specific nucleic acid stability in sewage, affected by environmental conditions, might affect comparative concentration estimates. PCR assay efficiency also differs between targets because of primer specificity, amplification conditions, and inhibition. Additionally, TAC detects all *V cholerae*, including outbreak strains O1 and O139, and the development of more specific cholera assays is ongoing. Clinical data encompassed individuals across Dhaka, whereas sewage surveillance covered a smaller area. More frequent, systematic sampling will be needed to strengthen the understanding of how environmental surveillance complements clinical systems and informs public health action.

Although no strong temporal variation of clinically significant AMR genes was detected, the mere presence of multiple AMR genes in sewage is concerning. Our findings align with those of previous reports that identified clinically significant AMR genes, including those encoding metallo-β-lactamases, in the sewage in Dhaka and in similar settings.^26^, ^27^, ^28^ Based on previous studies,^29^^,^^30^ two clinically relevant AMR genes in Bangladesh (*bla*_NDM_ and *mphA*) were found in our environmental surveillance. The co-occurrence of AMR genes in wastewater and clinical samples suggests that sewage surveillance might indicate community AMR burden and represent a reservoir for potential horizontal gene transfer. TACs enabled the rapid, broad detection of AMR genes across mechanisms and classes in both environmental and clinical samples. Further research is needed to quantify the links between environmental and clinical AMR gene presence in Dhaka.

The end date of this study coincided with the beginning of the SARS-CoV-2 pandemic. The establishment and validation of our environmental surveillance sites for this polio study led to the continuation and expansion of environmental surveillance in Dhaka to monitor SARS-CoV-2. This expanded surveillance activity tracked the pandemic in real time, showing that environmental surveillance provided an early warning of increases in transmission before clinical cases and evidence of persistent circulation in populations with limited access to clinical testing.^31^ Our findings were shared weekly with the Bangladeshi national COVID-19 task force for mitigation efforts via a weekly report and a dashboard that is publicly available.^32^ This use of environmental surveillance is a key example of how environmental surveillance sites that have been established for poliovirus surveillance can be quickly harnessed to provide surveillance for another pathogen with public health importance.

Although sewage surveillance for infectious diseases is increasing after the successful tracking of SARS-CoV-2 in sewage,^33^ the public health use case of sewage surveillance for other infectious diseases has not been formally established. TACs might be used to screen which pathogens are appropriate to include in integrated multipathogen sewage surveillance, and the public health use case should then be defined as well. TACs are modular and can be easily combined to create a new custom TAC with targets of interest. The ability to simultaneously detect more than 60 pathogens means it is one of the most cost-effective (US$63 per environmental surveillance sample: ∼US$1 per target in the proposed design compared with running 17 multiplex PCRs to cover 66 targets at US$5 per multiplex PCR), powerful molecular tools available. Moreover, the performance in terms of sensitivity and specificity is similar to that of other open-plate, higher-volume multiplex PCR.^12^ TACs also allow a more efficient, low-contamination workflow in the laboratory in terms of time to result (under 5 h), equipment needs, and required personnel. The proximity of the sampling sites to the laboratory allowed for a turnaround time of approximately 48 h from collection to laboratory results. Furthermore, TACs have produced reproducible and repeatable results across differing resourced laboratories.^12^

Direct poliovirus detection methods are being developed for current routine use by the GPEI to achieve eradication.^34^^,^^35^ Future research should identify which detection method will be used at which scale following eradication, when nearly all samples will be negative. One potential option would be to screen for poliovirus within integrated multipathogen surveillance with technology such as TACs (following further validation), and positive poliovirus samples could then be re-tested with genetic sequence-generating methods.

This study had some limitations. We did not compare the gold standard viral culture with TACs to detect poliovirus in stool or sewage samples in this population. Research is under way to validate the technology against the gold standard of poliovirus detection (cell culture and intratypic differentiation).^7^ Further validation of TACs is also required in different geographies (ie, both high-income and low-income settings) to capture formal and informal sewage networks with varying environmental conditions, and particularly for detecting circulating VDPV or wild poliovirus at low prevalence. The assessment of TACs for multipathogen surveillance also needs to be validated for a longer time period, with more frequent sampling. Nevertheless, the high concentration after the bOPV campaign provides important information, and our data will inform the design of future studies of multipathogen environmental surveillance.

In conclusion, the success of the GPEI to date is not only through effective vaccination but also through its establishment of a standardised surveillance system to track poliovirus. Maintaining a polio-free world following eradication will depend in part on designing an appropriate surveillance system that can be sustained without the large degree of international funding the programme has received to work towards its goal. Integrated sewage surveillance provides one such approach, and sensitive methods for this surveillance approach should be developed and validated.

## Data sharing

The sewage TAC qPCR data can be accessed through written request to the corresponding author with a proposal of secondary data analysis. After approval, data will be sent with a corresponding data dictionary.

## Declaration of interests

We declare no competing interests.
