# Supplementary material for: Integration of poliovirus and enteropathogen sewage surveillance in Dhaka Bangladesh: a longitudinal surveillance study, June 2019–June 2020
Source: Lancet Microbe. 2026 May;7(5):None. doi: 10.1016/j.lanmic.2025.101343 (PMC13197387; doi:10.1016/j.lanmic.2025.101343)
Supplement: Supplementary appendix [file mmc1.pdf]

THE LANCET
Microbe

Supplementary appendix

This appendix formed part of the original submission and has been peer reviewed. We post it as supplied by the authors.

Supplement to: Blake IM, Islam MO, Fuller B, et al. Integration of poliovirus and enteropathogen sewage surveillance in Dhaka Bangladesh: a longitudinal surveillance study, June 2019–June 2020. *Lancet Microbe* 2026. https://doi.org/10.1016/j.lanmic.2025.101343

Supplementary Materials

Integration of poliovirus and enteropathogen sewage surveillance in Dhaka, Bangladesh: a longitudinal surveillance study June 2019 – June 2020

Isobel M Blake[1](PhD), Md Ohedul Islam[2](MS), Benjamin Fuller[3](MD), Sarah Elwood[3](MS), Suporn Pholwat[3](PhD), Prof Jie Liu[3,4](PhD), Yoann Mira[5](MS), ASG Faruque[6](PhD), Firdausi Qadri[7](PhD), Rashidul Haque[7](PhD) and Prof Mami Taniuchi [2,3,8](PhD)

1. MRC Centre for Global Infectious Disease Analysis, School of Public Health, Imperial College London, UK

2. Department of Civil and Environmental Engineering, University of Virginia, Charlottesville, VA, USA

3. Division of Infectious Diseases and International Health, University of Virginia, Charlottesville, VA, USA

4. School of Public Health, Qingdao University, Qingdao, Shandong, China

5. Novel-T, Geneva, Switzerland

6. Nutrition Research Division,icddr,b, Dhaka, Bangladesh

7. Infectious Diseases Division, icddr,b, Dhaka, Bangladesh

8. Department of Biomedical Engineering, University of Virginia, Charlottesville, VA, USA

Supplementary Materials

Supplementary Methods Page 3

Table S1 Number of sewage samples collected per month per site Page 4

Table S2 TaqMan Array Card (TAC) target primer and probe sequences Page 5-7

Table S3 Univariable Hurdle Gamma Regression Model of Enterovirus detection in ES Samples Page 8

Table S4 Pearson correlation of monthly enteric pathogen concentration and clinical incidence Page 8

Figure S1 Physico-chemical measurements of the sewage in Wards 8, 9, 10 Page 9

Figure S2 QQplot of model residuals from the final multivariable mixed effects model of enterovirus detection from environmental surveillance in Dhaka. Page 10

Figure S3 Monthly mean concentration of those enteric pathogens without concurrent clinical data that were reliably detected from sewage samples collected in Wards 8, 9 and 10 of Dhaka, Bangladesh Page 10-11

Supplementary Text

BMFS grab sample collection and processing

Six-litre grab samples of wastewater are collected using the collection bag, sealed, and placed in clean buckets for transportation back to a field office. The sample was then filtered through ViroCap filters at the field office until the entire 6L passed through the filter or when 45 min had elapsed. Subsequently, the ViroCap filter housing was then placed on ice packs to maintain cold chain during transport to the icddr,b laboratory for further processing[1].

Pathogen elution, concentration, and total nucleic acid extraction

The pathogen was eluted using 1.5% beef extract, 0.05 M glycine, pH 9.5 eluent solution and further concentrated using skim milk flocculation[2]. Total nucleic acid (TNA) was extracted from the skim milk pellet using the QIAamp Stool Mini Kit (Qiagen, Gaithersburg, MD, USA) with a slightly modified manufacturer's protocol[3]. Extrinsic controls bacteriophage MS2 for the RNA targets and Phocine herpes virus (PhHV) for DNA targets were added to each sample during nucleic acid extraction process to monitor nucleic acid extraction and amplification efficiency[4]. An extraction blank was included per batch of extraction to monitor for contamination. The TNA was stored in -80°C until further testing.

Environmental Surveillance TaqMan Array Card (ES TAC)

Synthetic plasmid controls were used as positive control for validating the ES TAC. Synthetic plasmid controls contained each primer and probe sequences for all the targets. Limit of detection was 100 gene copies per reaction for each target. Validation of all assays were performed on analytical samples and sensitivity and specificity were 95-100% and 100%, respectively. On a limited number of positives from wastewater and clinical samples, specificity of the assays was confirmed by Sanger sequencing.

For each reaction mix, 20 µl of TNA sample was mixed with 50 µl of AgPath-ID One-Step RT-PCR buffer (2x; Applied Biosystems, Waltham, MA), 4 µl of AgPath-ID One-Step RT-PCR enzyme mix (25x; Applied Biosystems, Waltham, MA), and 26 µl of nuclease-free water to make a final volume of 100 µl. After thoroughly mixing the TNA sample with the reagents, the mixture was loaded into the assigned TAC card port. The card was centrifuged twice at 1200 rpm for 1 min, sealed properly, and the loading ports were excised. The card was then loaded into a ViiA7 or QuantStudio™ 7 Flex Real-Time PCR System (Applied Biosystems, Waltham, MA) for qPCR cycling as follows: 42°C for 45 min, 95°C for 3 min, followed by 15 cycles of 95°C for 24 s, 44°C for 30 s, 60°C for 24 s (ramp speed 0·25°C/s) and then 40 cycles of 95°C for 24 s, 47°C for 30 s and 65°C for 24 s (ramp speed 0·25°C/s, data collection step). The time to detection was 4 hours and 30 minutes.

Supplementary tables

Ward	Site	Jun 19	Jul 19	Aug 19	Sept 19	Oct 19	Dec 19	Jan 20	Feb 20	Mar 20	May 20	Jun 20
**8**	**1**	5	11	4	4	1	1	1	1	1	1	1
**8**	**2**	5	11	4	4	1	1	1	1	1	1	3
**8**	**3**	5	11	4	4	1	1	1	1	1	1	2
**8**	**4**	5	11	4	4	1	1	1	1	0	1	1
**9**	**1**	3	12	5	4	1	1	1	1	0	1	1
**9**	**2**	3	12	5	4	1	1	1	1	1	1	2
**9**	**3**	3	12	5	4	1	1	1	1	1	1	2
**9**	**4**	3	12	5	4	1	1	1	1	1	0	1
**10**	**1**	3	10	4	5	1	1	1	1	0	1	2
**10**	**2**	3	11	4	5	1	0	1	1	0	1	1
**10**	**3**	3	11	4	5	1	1	1	1	0	1	2
**10**	**4**	3	11	4	5	1	1	1	1	0	1	1

Table S1 Number of sewage samples collected per month per site in Dhaka, Bangladesh

Target Name	Forward Primer (5'→3')	Reverse Primer (5'→3')	Probe (5'→3')	Reference
**Sabin 1**	AGGTCAGATGCTTGAAAGC	CCACTGGCTTCAGTGTTT	FAM-CGCCCCCACCGTTTCACGGA-MGB	5
**Sabin 2**	CCGTTGAAGGGATTACTAAA	CGGCTTTGTGTCAGGCA	FAM-ATTGGTTCCCCCGACTTCCACCAAT-MGB	5
**Sabin 3**	AGGGCGCCCTAACTTT	TTAGTATCAGGTAAGCTATC	FAM-TCACTCCCGAAGCAACAG-MGB	5
**SOAS-WPV1**	CGTACAGACTAGRCAYGTNAT	GAGAATAAYTTGTCYTTKGAYGT	FAM-CATWATGGTTACRCAMGCACCT-MGB	5
**WEAF-WPV1**	GTACAAACCAGTCAYGTNAT	GAGAATAAYTTGTCYTTKGAYGT	FAM-CATWATGGTTACRCAMGCACCT-MGB	5
**PV2 (any serotype 2)-CDC**	GATGCAAAYAACGGICATGC	TCATAAAAGTGGGARTACGCRTT	FAM-ATGACTATACGTGGCAGAC-MGB	5
**PV2 (any serotype 2)-Modify**	CATGGAGTTCACYTTTGTGGTYA	TGTAAAAYACYGAYGGGTTAG	FAM-TAYATWCCACCHGGRGCACC-MGB	This study
**Africa WPV3-CDC**	CAGGGGGTTGATGAYTTRAT	ACKGTGTCTGAYGGNAC	FAM-CNCARAACAGYCTTCCGGATACC-MGB	5
**Africa WPV3-Modify**	CAGGGRGTYGAWGAYTTGAT	TTAGYGCTGGRACYTCCTTGG	FAM-CCAARCCACAAAACAGYCTT-MGB	This study
**South Asia WPV3-CDC**	GTYRTACARCGRCGYAGYAGRA	TCYTTRTAIGTRATGCGCCAAG	FAM-TTCTTYGCAAGIGGRGCRTGYGT-MGB	5
**South Asia WPV3-Modify**	TGCAAACACGBCAYGTCATA	TTYTGDGCYCTRGWBGTTGG	FAM-CRTGYGTYGCYATAATHGA-MGB	This study
**WPV3-Modify**	TGCAAACACGBCAYGTCAT	TTYTGDGCYCTRGWBGTTGG	FAM-CRTGYGTYGCYATAATHGA-MGB	This study
**Pan PV (any poliovirus)-CDC**	TTGGAGTTCTTCACITAITCIMGITTYGAYATG	GGAGCTCCGGGTGGGAYRTACATIATYTGRTAIAC	FAM-TGRTTNARIGCRTGICCRTTRTT-MGB	5
**Pan PV (any poliovirus)-Modify**	TTCNMGNTTTGAYATGGARTTYACHTT	CNCCNGGDGGKAYRTACATWAT	FAM-CHAAYAAYGGVCAYGC-MGB	This study
**Pan EV (any enterovirus)**	GGCCCCTGAATGCGGCTAATCC	GCGATTGTCACCATWAGCAGYCA	FAM-CCGACTACTTTGGGWGTCCGTGT-MGB	5
**MS2**	TGGCACTACCCCTCTCCGTATTCAC	GTACGGGCGACCCCACGATGAC	FAM-CACATCGATAGATCAAGGTGCCTACAAGC-MGB	17
**PhHV**	GGGCGAATCACAGATTGAATC	GCGGTTCCAAACGTACCAA	FAM-TATGTGTCCGCCACCATCT-MGB	16
**SHV**	TCCCATGATGAGCACCTTTAAA	TCCTGCTGGCGATAGTGGAT	FAM-TGCCGGTGACGAACAGCTGGAG-MGB	6
**SHV238-240SE-SK**	GTTGATCCGYTCCGTGCT	GCTTTGTTATKCGGGCCAAG	FAM-CGGAGCTAGCRARC-MGB	7
**mcr-1**	GATCGCTGTCGTGCTCTTTG	ACCGCGCCCATGATTAATAG	FAM-CGATGCTACTGATCACCACG-MGB	7
**mcr-2**	TGACATCACAKCACTCTTGGTATCG	TGTYGCYGCCAAAAAWAACG	FAM-CYTTTGTRCTGATGGGTTTGGT-MGB	7
**CTX-M1**	CCGTCACGCTGTTRTTAGGA	AATGCCACMCCCAGYCKKCC	FAM-CAGCAAAAACTTGCCGRATT-MGB	7
**CTX-M8-M25**	ATRACACSTTCCGGCTCGAT	GCTAAYGGCGTGGTGGTATC	FAM-TCAACACCGCGATCCCCG-MGB	8
**CTX-M2-M74**	GCGCAGACCCTGAAAAAYCT	TGYGCSCGCTGRGTTTCC	FAM-ACSCTGGGYAAAGCGC-MGB	7
**CTX-M9**	GCTTTATGCGCAGACGARTG	ATCACCGCGATAAAGCACCT	FAM-TCGATACCRMAGATAATACGC-MGB	7
**QnrA**	GGATTTGAGYGACAGYCGTTTT	CAATGAAACTGCAATCCTCGAA	FAM-CCGCTGCCGCTTYTATCA-MGB	7
**QnrS**	TTGCTCAGCMTTTATTWCWGGATGT	CAGCGATTTTCAWACARCTCACA	FAM-TATGCCAATATGGAGMGGGT-MGB	7
**QnrB1**	GYGGCGAGTTTWCGACTTT	TRGTCAGATCGCAATGTGTG	FAM-ACTGGCGAGCAGSRAACTT-MGB	7
**QnrB4**	ACVCGSACCTGGTTTTGYAG	GTACCCATCCAGCGGTTTTC	FAM-CTKGAAAAGTGCGARYTGT-MGB	7

**CMY1-MOX**	GCAACAACGACAATCCATCC	AARTAGTGGGCCTTGCCATC	FAM-CTGCTCAAGGAGCACAGGAT-MGB	7
**FOX**	CTGTGGACGGCATTATCCAG	TAGTGGGCYTTGCCATCTTT	FAM-ATGCTCAAGGAGTATCG-MGB	7
**CMY2-LAT**	GKHGGATTYGGCAGCTACGT	GYTYTTRTTTGCCARCATYA	FAM-TCRTTCCRGAAAAAAA-MGB	7
**ACT-MIR**	YGGRTTTGGCAGCTACKT	GCTTKTATTYGCSAGCATYACA	FAM-CTGCTTTTCRGGAATAAA-MGB	7
**aac(6')-lb-104W**	TATGCCCAGTCGTACGTTGC	GTTGTGATGCATTCGCCAGT	FAM-TCCCACCATCCGTCC-MGB	7
**aac(6')-lb-104R**	TATGCCCAGTCGTACGTTGC	GTTGTGATGCATTCGCCAGT	FAM-TCCCACCKTCCGTCC-MGB	7
**Aac(6')-lb-181Y**	CGATCCGATGCTACGAGAAAG	CCTGGCGTGTTTGAACCAT	FAM-CACCCCATATGGTCC-MGB	7
**KPC**	GGCCGCCGTGCAATAC	GCCGCCCAACTCCTTCA	FAM-TGATAACGCCGCCGCCAATTTGT-MGB	9
**DHA**	GYGCYTATGTSGCCTTTATTC	TCTTTCGGTATTCGGGTAGTTTTT	FAM-CAGGTGGCGATTGTGAT-MGB	7
**NDM**	ATATCACCGTTGGGATCGAC	TAGTGCTCAGTGTCGGCATC	FAM-AAGGACAGCAAGGCCAAGTCG-MGB	8
**VIM**	TSTACCCRTCCAATGGTCTC	AGAAGKGCCRCTGTGTTTTT	FAM-TGTCCGTGATGGYGATGAGTTG-MGB	8
**IMP**	AGTRGTTTGGYTRCCTRAAA	TKTGGCCAWGCTTYWAHATTTGC	FAM-TTYGGTGGTTGYTTTRTTAA-MGB	7
**OXA-48**	GCAAAGGAATGGCAAGAAAA	CACAACTACGCCCTGTGATTT	FAM-AGTTGGAATGCTCACTTTACTG-MGB	8
**gyrA 83F-Y (Sal)**	TGGTGACGTAATCGGTAAATACCA	ATCAGTTCGTGGGCGATTTT	FAM-TGCGWAATCGCC-MGB	7
**gyrA 87D (Sal.)**	TGGTGACGTAATCGGTAAATACCA	ATCAGTTCGTGGGCGATTTT	FAM-AGTGTATGACACCATC-MGB	7
**gyrA 87G (Sal.)**	TGGTGACGTAATCGGTAAATACCA	ATCAGTTCGTGGGCGATTTT	FAM-GTGTATGGCACCATC-MGB	7
**gyrA 87N-Y (Sal.)**	TGGTGACGTAATCGGTAAATACCA	ATCAGTTCGTGGGCGATTTT	FAM-GCAGTGTATWACACCAT-MGB	7
**parC 80I (Sal.)**	GACGTACTGGGTAAGTATCACCCG	ATCGCCGCGAATGACTTC	FAM-CAGGCGATGTCG-MGB	7
**gyrA 86I (C.jejuni)**	GCCCGTATAGTGGGTGCTGT	TCTTGAGCCATTCTAACCAAAGC	FAM-AACTGCTATATCTCC-MGB	7
**gyrA 86I (C.coli)**	TCTGCTCGTATAGTAGGGGATGTT	GCATAGAGAAATCTTGTGCCATTC	FAM-ACAGCAATATCGC-MGB	7
**QepA**	TGTTCGGCGTCTACATCTTCA	GAACCGATGACGAAGCACAG	FAM-GACGCAGTACCTGCAGCTC-MGB	7
**23S-2075G (Campy)**	GATCCAGTGAAATTGTAGTGGAGGT	GGCTCATATACAACTGGCGTCATA	FAM-GACGGAGAGACCC-MGB	7,10
**ermB**	CGTACCTTGGATATTCACCG	GTAAACAGTTGACGATATTCTCG	FAM-TGCACACTCAAGTCTCGATTCAGC-MGB	11
**mphA**	GTGCTGGCAATGCTCAAGAA	TGACCATCGCAGTCGAGTCT	FAM-AGCTCGTTGCCTATCCCAT-MGB	7
**Enterobacteriaceae 16S**	GGGGGTAGAATTCCAGGTGT	CAAGGGCACAACCTCCAAG	FAM-TGGGGAGCAAACAGGATTAG-MGB	7
**Giardia**	GACGGCTCAGGACAACGGTT	TTGCCAGCGGTGTCCG	FAM-CCCGCGGCGGTCCCTGCTAG-MGB	12
**Pan-Campy**	AAAGTIGGMAAAGATGGTGTTAT<br>AAAGTIGGWAAAGACGGYGTTAT	TCAAATTGCATACCYTCAAC	FAM-TTTGCCTCTTCMACAGT-MGB	19
**Cryptosporidium**	GGGTTGTATTTATTAGATAAAGAACCA	AGGCCAATACCCTACCGTCT	FAM-TGACATATCATTCAAGTTTCTGAC-MGB	16
**Shigella spp.**	CCTTTTCCGCGTTCCTTGA	CGGAATCCGGAGGTATTGC	FAM-CGCCTTTCCGATACCGTCTCTGCA-MGB	13

**Salmonella spp.**	CTCACCAGGAGATTACAACATGG	AGCTCAGACCAAAAGTGACCATC	FAM-CACCGACGGCGAGACCGACTTT-MGB	14
**C.jejuni-coli**	CWGCTAAACCATARAAATAAAATTTCTCAC	YTTTGAAGGTAATTTAGATATGGATAATCG	FAM-CATTTTGAYGATTTTTGGCTTGA-MGB	15
**S.enteriditis**	GGTGGCTGGCGAATGGT	GATTCAGGGAGTATATCAAAAAGGTTTAG	FAM-AGCAGACAACAGGCTG-MGB	This study
**S.typhimurium**	CTGACAGACGCGGTCAAATAA	GTGAACACCTGAAGTATCTGTTG	VIC-CGTCGACATGCTCACTG-MGB	This study
**S.paratyphi A**	GCGGGGAACACGAATCATTC	GCATCATCGCGCATAGTGTC	FAM-CTCGGTTTATCCCCGCTGG-MGB	20
**S.typhi**	CGCGAAGTCAGAGTCGACATAG	AAGACCTCAACGCCGATCAC	FAM-CAGCCTGCTCCAGAACA-MGB	18
**EAEC(aatA)**	CTGGCGAAAGACTGTATCAT	TTTTGCTTCATAAGCCGATAGA	FAM-TGGTTCTCATCTATTACAGACAGC-MGB	16
**EAEC(aaiC)**	ATTGTCCTCAGGCATTTCAC	ACGACACCCCTGATAAACAA	FAM-TAGTGCATACTCATCATTTAAG-MGB	16

Table S2 Environmental Surveillance TaqMan Array Card (TAC) target primer and probe sequences

Variable	Odds Ratio (95% CI) associated with a negative sample	Rate Ratio of viral load, conditional of the sample being positive
**pH**	0.91 (0.19, 4.47)	2.52 (0.93, 6.81)
**ORP, per absolute increase of 100 units (mV)**	0.94 (0.61, 1.43)	1.84 (1.4, 2.42)
**Dissolved Oxygen (mg/L)**	1.02 (0.85, 1.24)	1.08 (0.98, 1.19)
**Log site population density**	1.07 (0.51, 2.22)	1.12 (0.59, 2.12)
**TDS, per absolute increase of 100 units (mg/L)**	0.77 (0.66, 0.89)	0.99 (0.9, 1.09)
**First 21 days following immunisation campaign with bivalent oral polio vaccine cf rest of study period**	0.46 (0.27, 0.79)	1.7 (1.3, 2.23)
**Sewage Temperature, per degree increase**	0.83 (0.72, 0.95)	0.93 (0.85, 1.02)
**Site watershed area (km2)**	1.09 (0.84, 1.41)	0.9 (0.73, 1.11)
**Log of site catchment population (children under 5 years old)**	1.18 (0.89, 1.56)	0.86 (0.68, 1.08)
**Reported coverage of receiving 3 Oral Polio Vaccine doses**	0.37 (0, 3271.75)	1.64 (0, 4360.14)
**Proportion of catchment reporting having a sanitary toilet with no flush**	0.76 (0.04, 13.66)	1.99 (0.18, 22.37)

Table S3 Univariable Hurdle Gamma Regression Model of Enterovirus detection in ES Samples. The variable was either included in the Hurdle model or the Gamma model.

Pathogen	Pearson Correlation (p value)
**Rotavirus**	0.828 (p = 0.002)
V. cholerae	0.085 (p = 0.804)
**Salmonella**	-0.055 (p = 0.874)
**ETEC**	-0.280 (p = 0.404)
**Shigella**	0.245 (p = 0.467)

Table S4 Pearson linear correlation between mean monthly enteric pathogen concentration and monthly clinical incidence at icddr,b hospital for each respective pathogen

Supplementary Figures

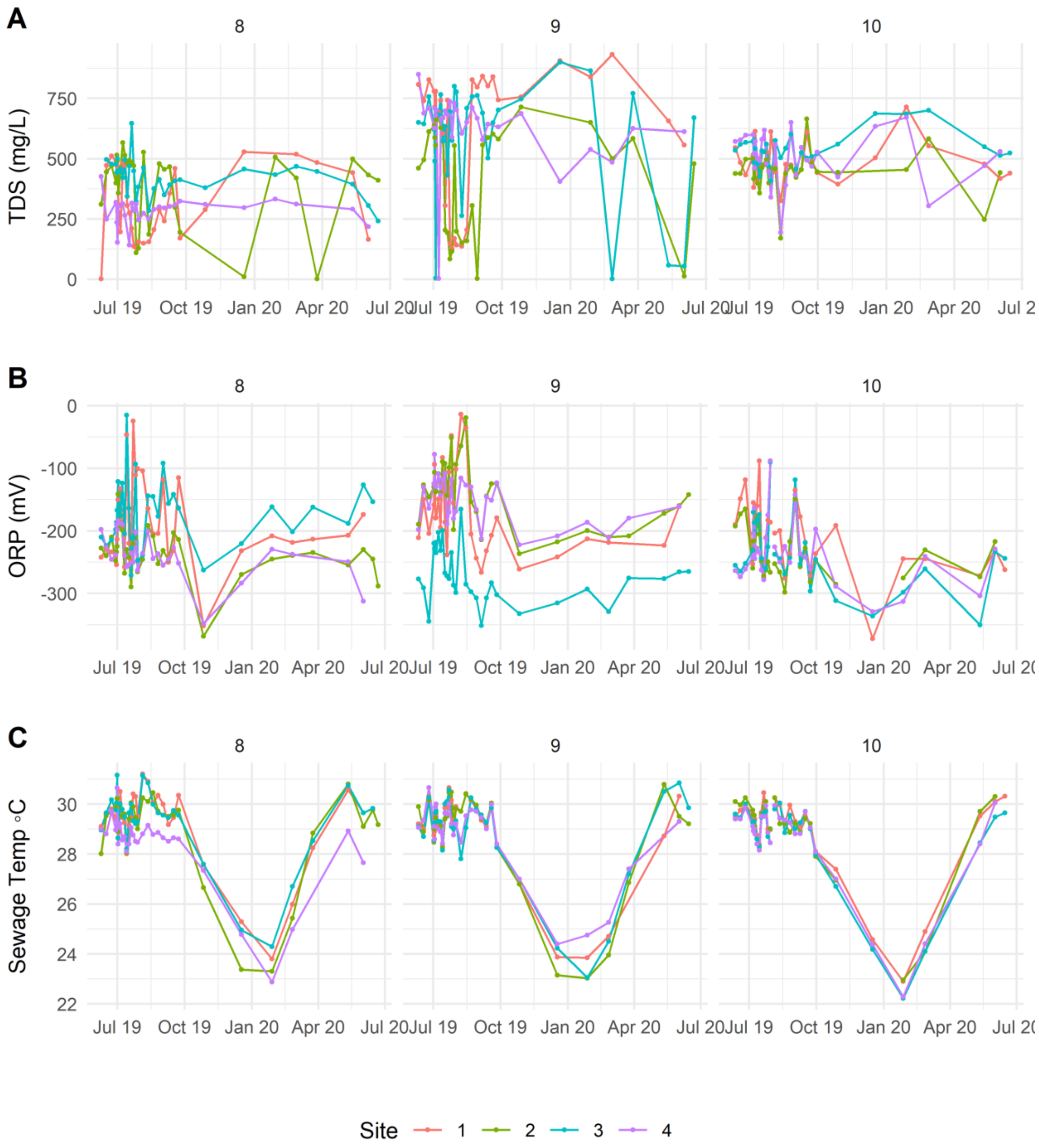

Figure S1 Physico-chemical measurements of the sewage in Wards 8, 9, 10 at the time of sampling. TDS: Total dissolved solids. ORP: Oxidative Reduction Potential.

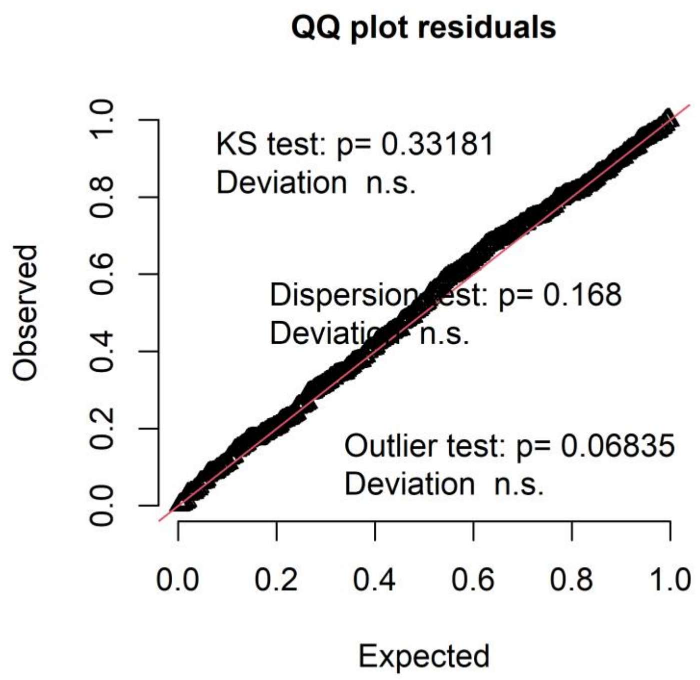

Figure S2 QQplot of model residuals from the final multivariable mixed effects model of enterovirus detection from environmental surveillance in Dhaka. Residuals were calculated using the DHARMa package[21] and running 10,000 model simulations.

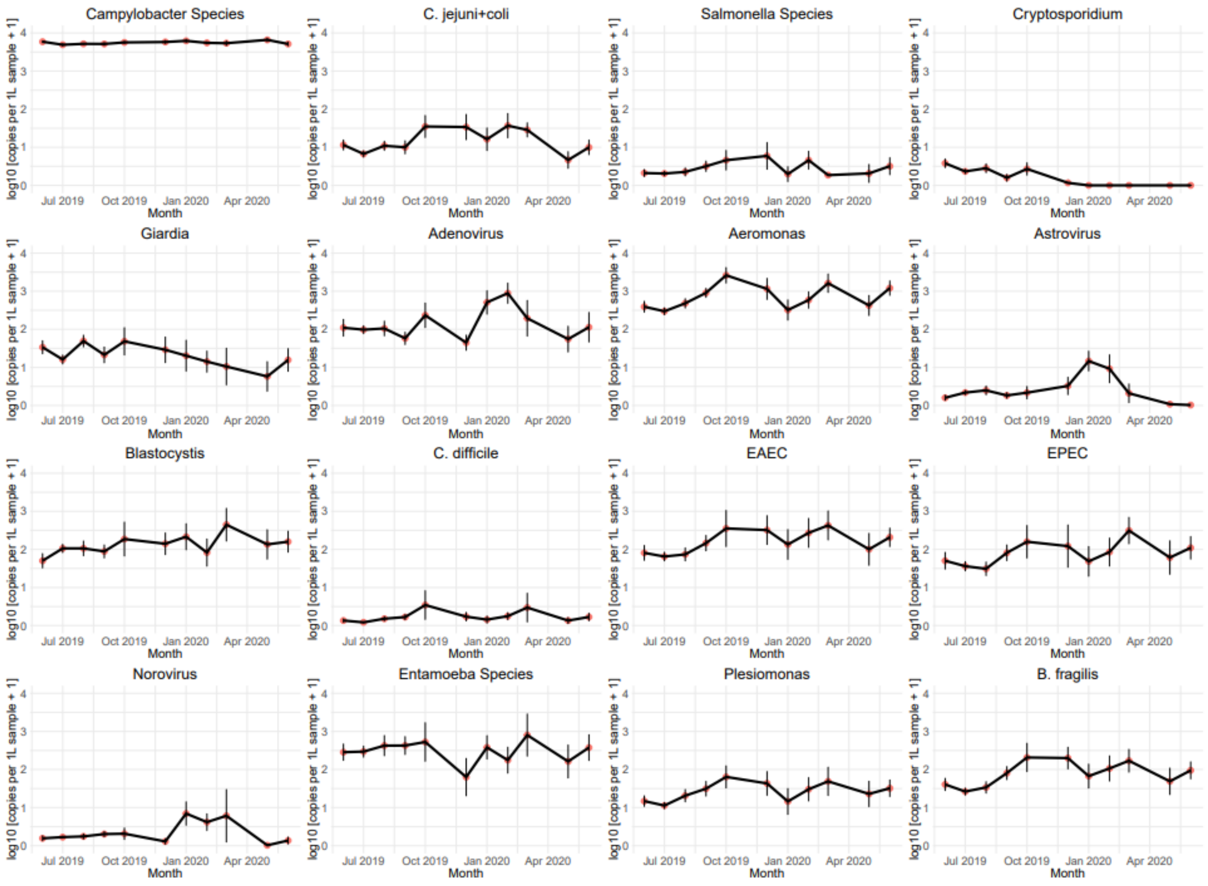

Figure S3 Monthly mean concentration of those enteric pathogens without concurrent clinical data that were reliably detected from sewage samples collected in Wards 8, 9 and 10 of Dhaka, Bangladesh (error bars denote 95% CI around the mean).

References

1. Rogawski McQuade ET, Blake IM, Brennhofer SA, Islam MO, Sony SSS, Rahman T, et al. Real-time sewage surveillance for SARS-CoV-2 in Dhaka, Bangladesh versus clinical COVID-19 surveillance: a longitudinal environmental surveillance study (December, 2019-December, 2021). Lancet Microbe. 2023 Jun;4(6):e442–51.

2. Liu J, Gratz J, Maro A, Kumburu H, Kibiki G, Taniuchi M, et al. Simultaneous detection of six diarrhea-causing bacterial pathogens with an in-house PCR-luminex assay. J Clin Microbiol. 2012 Jan;50(1):98–103.

3. Liu J, Kabir F, Manneh J, Lertsethtakarn P, Begum S, Gratz J, et al. Development and assessment of molecular diagnostic tests for 15 enteropathogens causing childhood diarrhoea: a multicentre study. Lancet Infect Dis. 2014 Aug;14(8):716–24.

4. Liu J, Gratz J, Amour C, Nshama R, Walongo T, Maro A, et al. Optimization of Quantitative PCR Methods for Enteropathogen Detection. PLOS ONE. 2016 Jun 23;11(6):e0158199.

5. Gerloff N, Sun H, Mandelbaum M, Maher C, Nix WA, Zaidi S, Shaukat S, Seakamela L, Nalavade UP, Sharma DK, Oberste MS, Vega E. Diagnostic Assay Development for Poliovirus Eradication. J Clin Microbiol. 2018 Jan 24;56(2):e01624-17. doi: 10.1128/JCM.01624-17. PMID: 29212703; PMCID: PMC5786708.

6. Roschanski N, Fischer J, Guerra B, Roesler U. Development of a multiplex real-time PCR for the rapid detection of the predominant beta-lactamase genes CTX-M, SHV, TEM and CIT-type AmpCs in Enterobacteriaceae. PLoS One. 2014 Jul 17;9(7):e100956. doi: 10.1371/journal.pone.0100956. PMID: 25033234; PMCID: PMC4102473.

7. Pholwat S, Liu J, Taniuchi M, Chinli R, Pongpan T, Thaipisutikul I, Ratanakorn P, Platts-Mills JA, Fleece M, Stroup S, Gratz J, Mduma E, Mujaga B, Walongo T, Nshama R, Kimathi C, Foongladda S, Houpt ER. Genotypic antimicrobial resistance assays for use on E. coli isolates and stool specimens. PLoS One. 2019 May 10;14(5):e0216747. doi: 10.1371/journal.pone.0216747. PMID: 31075137; PMCID: PMC6510447.

8. Chavda KD, Satlin MJ, Chen L, Manca C, Jenkins SG, Walsh TJ, Kreiswirth BN. Evaluation of a Multiplex PCR Assay To Rapidly Detect Enterobacteriaceae with a Broad Range of β-Lactamases Directly from Perianal Swabs. Antimicrob Agents Chemother. 2016 Oct 21;60(11):6957-6961. doi: 10.1128/AAC.01458-16. PMID: 27600053; PMCID: PMC5075117.

9. Centers for Disease Control and Prevention. 2011. Multiplex real-time PCR detection of Klebsiella pneumoniae carbapenemase (KPC) and New Delhi metallo-β-lactamase (NDM-1). https://www.cdc.gov/gram-negative-bacteria/php/laboratories/?CDC_AAref_Val=https://www.cdc.gov/HAI/settings/lab/kpc-ndm1-lab-protocol.html.

10. Hao H, Liu J, Kuang X, Dai M, Cheng G, Wang X, Peng D, Huang L, Ahmad I, Ren N, Liu Z, Wang Y, Yuan Z. Identification of Campylobacter jejuni and determination of point mutations associated with macrolide resistance using a multiplex TaqMan MGB real-time PCR. J Appl Microbiol. 2015 Jun;118(6):1418-25. doi: 10.1111/jam.12793. Epub 2015 Apr 14. PMID: 25766481.

11. Kumari N, Navaratnam P, Sekaran SD. Detection of pbp2b and ermB genes in clinical isolates of Streptococcus pneumoniae. J Infect Dev Ctries. 2008 Jun 1;2(3):193-9. doi: 10.3855/jidc.262. PMID: 19738350.

12. Verweij JJ, Schinkel J, Laeijendecker D, van Rooyen MA, van Lieshout L, Polderman AM. Real-time PCR for the detection of Giardia lamblia. Mol Cell Probes. 2003 Oct;17(5):223-5. doi: 10.1016/s0890-8508(03)00057-4. PMID: 14580396.

13. Vu DT, Sethabutr O, Von Seidlein L, Tran VT, Do GC, Bui TC, Le HT, Lee H, Houng HS, Hale TL, Clemens JD, Mason C, Dang DT. Detection of Shigella by a PCR assay targeting the ipaH gene suggests increased prevalence of shigellosis in Nha Trang, Vietnam. J Clin Microbiol. 2004 May;42(5):2031-5. doi: 10.1128/JCM.42.5.2031-2035.2004. PMID: 15131166; PMCID: PMC404673.

14. Malorny B, Paccassoni E, Fach P, Bunge C, Martin A, Helmuth R. Diagnostic real-time PCR for detection of Salmonella in food. Appl Environ Microbiol. 2004 Dec;70(12):7046-52. doi: 10.1128/AEM.70.12.7046-7052.2004. PMID: 15574899; PMCID: PMC535175.

15. Cunningham SA, Sloan LM, Nyre LM, Vetter EA, Mandrekar J, Patel R. Three-hour molecular detection of Campylobacter, Salmonella, Yersinia, and Shigella species in feces with accuracy as high as that of culture. J Clin Microbiol. 2010 Aug;48(8):2929-33. doi: 10.1128/JCM.00339-10. Epub 2010 Jun 2. Erratum in: J Clin Microbiol. 2011 Oct;49(10):3725. PMID: 20519461; PMCID: PMC2916566.

16. Liu J, Gratz J, Amour C, Kibiki G, Becker S, Janaki L, Verweij JJ, Taniuchi M, Sobuz SU, Haque R, Haverstick DM, Houpt ER. A laboratory-developed TaqMan Array Card for simultaneous detection of 19 enteropathogens. J Clin Microbiol. 2013 Feb;51(2):472-80. doi: 10.1128/JCM.02658-12. Epub 2012 Nov 21. PMID: 23175269; PMCID: PMC3553916.

17. Rolfe KJ, Parmar S, Mururi D, Wreghitt TG, Jalal H, Zhang H, Curran MD. An internally controlled, one-step, real-time RT-PCR assay for norovirus detection and genogrouping. J Clin Virol. 2007 Aug;39(4):318-21. doi: 10.1016/j.jcv.2007.05.005. Epub 2007 Jun 28. PMID: 17604686.

18. Liu J, Ochieng C, Wiersma S, Ströher U, Towner JS, Whitmer S, Nichol ST, Moore CC, Kersh GJ, Kato C, Sexton C, Petersen J, Massung R, Hercik C, Crump JA, Kibiki G, Maro A, Mujaga B, Gratz J, Jacob ST, Banura P, Scheld WM, Juma B, Onyango CO, Montgomery JM, Houpt E, Fields B. Development of a TaqMan Array Card for Acute-Febrile-Illness Outbreak Investigation and Surveillance of Emerging Pathogens, Including Ebola Virus. J Clin Microbiol. 2016 Jan;54(1):49-58. doi: 10.1128/JCM.02257-15. Epub 2015 Oct 21. PMID: 26491176; PMCID: PMC4702733.

19. Liu J, Gratz J, Amour C, Nshama R, Walongo T, Maro A, Mduma E, Platts-Mills J, Boisen N, Nataro J, Haverstick DM, Kabir F, Lertsethtakarn P, Silapong S, Jeamwattanalert P, Bodhidatta L, Mason C, Begum S, Haque R, Praharaj I, Kang G, Houpt ER. Optimization of Quantitative PCR Methods for Enteropathogen Detection. PLoS One. 2016 Jun 23;11(6):e0158199. doi: 10.1371/journal.pone.0158199. PMID: 27336160; PMCID: PMC4918952.

20. Rainey JJ, Siesel C, Guo X, Yi L, Zhang Y, Wu S, Cohen AL, Liu J, Houpt E, Fields B, Yang Z, Ke C. Etiology of acute febrile illnesses in Southern China: Findings from a two-year sentinel surveillance project, 2017-2019. PLoS One. 2022 Jun 28;17(6):e0270586. doi: 10.1371/journal.pone.0270586. PMID: 35763515; PMCID: PMC9239456.

21. Hartig F (2024). _DHARMa: Residual Diagnostics for Hierarchical (Multi-Level / Mixed) Regression Models_. doi:10.32614/CRAN.package.DHARMa <https://doi.org/10.32614/CRAN.package.DHARMa>, R package version 0.4.7.
